# Hippo/YAP signaling’s multifaceted crosstalk in cancer

**DOI:** 10.3389/fcell.2025.1595362

**Published:** 2025-07-02

**Authors:** Jie Zhang, Haipeng Wu, Xinxin Ren, Zhuoshi Chen, Siyu Ye, Shuchang Chen, Jie Fang, Qirou Wu, Tiejun Zhao

**Affiliations:** ^1^ Key Laboratory of Novel Targets and Drug Study for Neural Repair of Zhejiang Province, School of Medicine, Hangzhou City University, Hangzhou, China; ^2^ College of Pharmaceutical Science, Zhejiang University, Hangzhou, China; ^3^ College of Life Sciences, Zhejiang Normal University, Jinhua, China

**Keywords:** Hippo/YAP signaling, signaling pathway, crosstalk, cancer, therapeutic target

## Abstract

The Hippo/yes-associated protein (YAP) signaling is an evolutionarily conserved regulator in organ size control, which plays pivotal roles in cell proliferation, differentiation, apoptosis, and tissue regeneration. In cancer, dysregulation of Hippo/YAP signaling is typically recognized as one of the crucial drivers in tumorigenesis. However, beyond its canonical transcriptional targets, Hippo/YAP signaling engages in extensive crosstalk with multiple pathways to form an intricate regulatory network, thereby giving rise to its content-dependent influence on tumor initiation, progression and metastasis. This review focuses on the molecular mechanisms underlying the interplay between Hippo/YAP and pivotal signaling pathways such as nuclear factor kappa-light-chain-enhancer of activated B cells (NF-κB), wingless-type (Wnt)/β-catenin signaling pathway, transforming growth factor-beta (TGF-β), Hedgehog, Notch and other signaling pathways, as well as their implications in cancer biology. Ultimately, exploiting these mechanisms may represent promising therapeutic strategies for cancer.

## 1 Introduction

The Hippo/YAP signaling is an evolutionarily conserved kinase cascade, with its core effectors YAP and transcriptional co-activator with PDZ-binding motif (TAZ) playing critical roles in the regulation of organ size, cell proliferation, differentiation, apoptosis, and tissue regeneration (Franklin et al., 2023). Under normal physiological conditions, YAP/TAZ are phosphorylated by large tumor suppressor kinase 1/2 (LATS1/2) and retained in the cytoplasm. Upon Hippo/YAP pathway inactivation, they translocate into the nucleus and partner with TEA domain transcription factors (TEADs) to activate various target genes such as connective tissue growth factor (CTGF) and cysteine-rich angiogenic protein 61 (CYR61) to promote cellular growth and survival (Yao et al., 2024). This dynamic nuclear-cytoplasmic shuttling of YAP/TAZ is tightly regulated by mechanisms like post-translational modification and nuclear import/export machinery (Kofler et al., 2018). In cancer, the overexpression of YAP/TAZ is frequently observed in diverse malignancies with poor clinical outcomes, including breast, colorectal, and liver cancers (Chen et al., 2023; Liu et al., 2022). Besides, dysregulations of this pathway, characterized by sustained nuclear accumulation and transcriptional hyperactivation of YAP/TAZ, perturbs the tightly controlled equilibrium between cell proliferation and apoptosis, thereby driving tumor initiation, malignant progression, metastatic dissemination, and the development of therapeutic resistance (Harvey et al., 2013; Luo et al., 2023).

Recent studies have revealed that Hippo/YAP signaling not only regulate the canonical downstream genes related to proliferation, but also engage in extensive crosstalk with multiple signaling pathways. The crosstalk is dynamic and involves pathways such as NF-κB (Govorova et al., 2024), Wnt/β-catenin (Jiang et al., 2020), TGF-β/Smad (Piccolo et al., 2023), Hedgehog (Sharma et al., 2022), Notch (Totaro et al., 2018a), mechanistic target of rapamycin (mTOR) (Yang et al., 2020), and immune checkpoint axes (Miao et al., 2017). For example, microRNA-301a mediates crosstalk between Hedgehog and Hippo/YAP signaling to promote pancreatic ductal adenocarcinoma progression (Qi et al., 2025). Meanwhile, YAP/TAZ-driven immune evasion via programmed death-ligand 1 (PD-L1) upregulation underscores the interface between Hippo signaling and immune checkpoints (Nguyen and Yi, 2019). The regulatory network centered on Hippo/YAP signaling has a bidirectional role that is highly context-dependent. For example, in pancreatic ductal adenocarcinoma, the ubiquitination of mammalian Ste20-like kinase 1 (MST1) by TNF receptor-associated factor 6 (TRAF6) unleashes YAP activity to amplify inflammation, while YAP overexpression disrupts the interaction of TGF-β-activated kinase 1 (TAK1) and inhibitor of nuclear factor kappa-B kinase subunit beta (IKKβ) to attenuate NF-κB pathway and mitigate inflammation (Wang et al., 2020).

Although numerous studies have explored the interactions between Hippo/YAP and individual signaling cascades, the comprehensive integrative analyses that consolidate these interconnections into a unified regulatory network remains limited. In this review, we summarize recent advances in understanding the complex signaling crosstalk between Hippo/YAP signaling and major oncogenic pathways. We further discuss the regulatory details in the mechanism, and the contextual determinants of these interactions, such as subcellular localization, co-factor availability, and chromatin accessibility (Zheng et al., 2025). Finally, we highlight the emerging therapeutic strategies targeting Hippo/YAP signaling and combinatorial approaches involved (Papavassiliou et al., 2023; Zhao et al., 2023).

## 2 Hippo/YAP signaling

**FIGURE 1:**
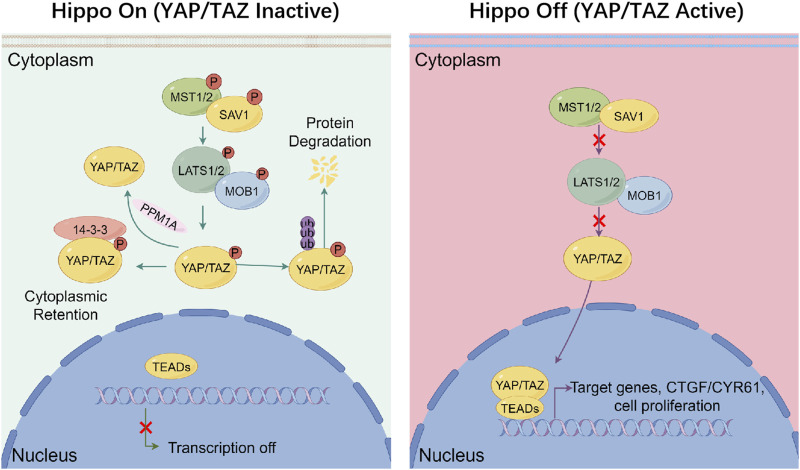
Canonical regulation of hippo signaling. Under Hippo On conditions (left), phosphorylated upstream kinases MST1/2 form a complex with SAV1 to phosphorylate and activate LATS1/2–MOB1 complex. Activated LATS1/2 phosphorylates the co-activators YAP and TAZ, leading to their cytoplasmic retention via binding to 14-3-3 proteins and subsequent degradation through proteasome, thereby preventing their nuclear translocation and transcriptional activity. The phosphatase PPM1A can counteract this process by dephosphorylating YAP/TAZ. In contrast, under Hippo Off conditions (right), the kinase cascade is inactive, and YAP/TAZ remain unphosphorylated. This allows them to accumulate in the nucleus, where they bind to TEAD transcription factors and promote the expression of genes like CTGF and CYR61, which involved in cell proliferation and survival. This signaling switch integrates inputs from mechanical stress, cell polarity, and oncogenic signals to maintain tissue homeostasis.

The Hippo/YAP signaling, originally identified in *Drosophila* through genetic mosaic screening, constitutes an evolutionarily conserved kinase cascade that governs organ size by restricting the activity of the transcriptional co-activator Yorkie (Yki). In mammals, the functional homologues of Yki are YAP and TAZ (Huang et al., 2005; Fu et al., 2022). This kinase cascade commences with the activation of MST1/2, which subsequently phosphorylate and activate the LATS1/2 with the scaffold protein salvador homolog 1 (SAV1). Once activated, LATS1/2 phosphorylate the downstream effectors YAP/TAZ with the scaffold protein mps one binder 1 (MOB1), leading to the interactions with 14-3-3 protein and degradation through proteasome in cytoplasm (Zhang et al., 2015). The phosphatase magnesium-dependent 1A (PPM1A) and pre-mRNA processing factor kinase (PRP4K) mediate YAP/TAZ dephosphorylation via distinct mechanisms, thereby finely regulating its intracellular localization (Zhou et al., 2021; Cho et al., 2018). Besides, the disruption of upstream regulatory inputs, including loss of thousand-and-one amino acid kinase (TAO1/2/3) or moesin-ezrin-radixin-like protein (NF2/Merlin) function, abrogates MST1/2 and LATS1/2 activation, culminating in the nuclear accumulation of dephosphorylated YAP/TAZ, association with TEAD transcription factors, and the induction of gene expression programs that drive cellular proliferation and migration (Figure 1) (Totaro et al., 2018b).

YAP/TAZ are typically recognized as oncogenic drivers in a broad spectrum of human cancers. Increased activity and nuclear localization of YAP/TAZ promotes tumor cell proliferation, migration, and resistance to apoptosis through classical transcriptional regulation, while also promoting tumor growth via non-cell-autonomous mechanisms, including the secretion of pro-angiogenic factors and suppression of immune responses (Baroja et al., 2024). Dysregulation of Hippo/YAP signaling has also been implicated in the therapy resistance like chemoresistance in non-small cell lung cancer and triple-negative breast cancer, suggesting that YAP/TAZ represent promising targets for combination therapeutic strategies (Chapeau et al., 2024). Besides, in breast cancer, YAP/TAZ activity is positively correlated with transaminase expression levels in patients. Evidence has shown that YAP/TAZ reprograms cellular energetics to promote the dependence of breast cancer cell growth on exogenous glutamine (Yang et al., 2018). Furthermore, YAP and TAZ regulate glutamine metabolism by influencing the expression of key enzymes and transporters, contributing to tumor progression (Edwards et al., 2017).

However, recent findings indicate that Hippo/YAP signaling can also act as a tumor suppressor in a context-dependent manner. In colorectal cancer and acute myeloid leukemia, the activation of YAP paradoxically induces cell death or stimulate anti-tumor immunity (Baroja et al., 2024). And in estrogen receptor alpha (ERα) positive breast cancer, YAP upregulates vestigial like family member 3 (VGLL3), recruiting the nuclear receptor co-repressor (NCOR2/SMRT) repressor to the estrogen receptor 1 (ESR1) super-enhancer, thereby silencing ERα transcription and inhibiting tumor growth (Ma et al., 2022). Independently, MST1/2 inhibition or YAP activation promotes the proteasomal degradation of ERα, blocks both hormone-dependent and independent ERα transcriptional programs, overcoming ESR1 mutant-driven resistance (Li et al., 2022). YAP-mediated tumor-suppressive effects are also detected in resistant models like androgen receptor (AR) in prostate cancer. Elevated nuclear YAP displaces AR from TEAD complexes, attenuates AR target gene transcription to suppresses growth of both full-length and therapy-resistant AR variant tumors (Li et al., 2023). Comprehensive analyses caution that systemic Hippo inhibition may yield unpredictable outcomes across tumor types, advocating for context-informed therapeutic strategies (Luo et al., 2023).

Beyond its role in tumorigenesis, the Hippo/YAP signaling also contributes to various pathological conditions, including cardiovascular diseases, neurological disorders, immune system dysfunctions, trauma, and chronic inflammatory diseases (Ajongbolo and Langhans, 2025). These findings highlight the multifaceted nature of the Hippo/YAP signaling and its extensive therapeutic potential for addressing a wide range of pathological conditions (Harvey et al., 2003).

## 3 Hippo/YAP and NF-κB pathway

The NF-κB pathway regulates multiple aspects of innate and adaptive immunity and serves as a key mediator in inflammatory responses. The key factors of NF-κB pathway are v-rel reticuloendotheliosis viral oncogene homolog (REL) family members, including nuclear factor kappa B subunit RelA (p65), RelB proto-oncogene (RelB), v-rel reticuloendotheliosis viral oncogene homolog (c-Rel), nuclear factor kappa B subunit 1 (p50/p105), and nuclear factor kappa B subunit 2 (p52/p100) (Guo et al., 2024). Upon activation through either canonical or non-canonical signalings, NF-κB translocates into the nucleus and directs extensive inflammation-associated transcriptional reprogramming (Dolcet et al., 2005).

Various evidence indicates that YAP functions as a transcriptional co-activator of NF-κB, reinforcing inflammatory and oncogenic signals in various cancers. For example, in osteoclastogenesis, YAP enhances NF-κB pathway to drive osteoclast differentiation and bone resorption, facilitating skeletal metastasis in cancer (Zhao et al., 2018). In soft tissue sarcoma, YAP inhibits the expression of ubiquitin specific peptidase 31 (USP31), a key upstream negative regulator of NF-κB, thereby diminishing its inhibitory effect on NF-κB activity (Ye et al., 2018). And in endometrial cancer, YAP enhances cell proliferation, migration, and invasion by upregulating interleukin-11 (IL-11) transcription through p65, exacerbating the malignancy (Guo et al., 2021). However, emerging evidence indicates that the Hippo/YAP signaling plays dual roles in regulating NF-κB pathway in a context-dependent manner. In certain cases like clear cell renal cell carcinoma (ccRCC), YAP represses the TEAD-NF-κB complex by competing with TEAD for p65 binding, preventing p65 recruitment to target gene promoters to attenuate inflammation-driven tumorigenesis (Li et al., 2024). Additionally, YAP inhibits NF-κB pathway and ccRCC growth by opposing zinc fingers and homeoboxes protein 2 (ZHX2), a critical p65 co-activator, further demonstrating its tumor-suppressive role in ccRCC (Li et al., 2025).

The crosstalk of Hippo/YAP and NF-κB pathway is influenced by multiple factors, including the subcellular localization of YAP. When localized in the nucleus, YAP tends to function as a co-activator of p65, enhancing NF-κB transcriptional activity, while cytoplasmic YAP often stabilizes IkBa or facilitates TRAF6 degradation, thereby dampening NF-κB pathway (Kong et al., 2024). Additionally, synergistic effects are more frequently observed in bone, kidney, and gynecologic tumors, whereas endothelial and epithelial cells often exhibit antagonistic effects. Besides, the composition of the transcriptional co-activator complex, such as the presence of TEAD, histone deacetylase 7 (HDAC7), or mediator complex subunit 2 (MED2), further modulates the genomic targeting and functional output of YAP-p65 complexes (Kong et al., 2024; Lopez-Hernandez et al., 2021).

Additionally, recent studies demonstrate that the NF-κB pathway can also influence the Hippo/YAP signaling (Caire et al., 2022). Activation of NF-κB pathway induces YAP nuclear translocation and enhances its transcriptional activity to promote cell survival and modulate the expression of genes involved in inflammatory and fibrotic pathways. Notably, YAP was found to upregulate tumor necrosis factor receptor-associated factor 2 (TRAF2) expression (Caire et al., 2022), a key adaptor in TNF receptor signaling, suggesting a mechanism by which YAP amplifies NF-κB-mediated inflammatory responses. Moreover, additional regulatory factors, such as long non-coding RNA (lncRNA) or unidentified scaffolding proteins, may influence the crosstalk of Hippo/YAP and NF-κB pathway (Xu et al., 2021). Integrative single-cell multi-omics approaches, coupled with chromatin immunoprecipitation sequencing (ChIP-seq) and assay for transposase-accessible chromatin using sequencing (ATAC-seq), will be essential for delineating YAP-target networks in diverse tumors (Yu et al., 2023), offering mechanistic insights and new opportunities for targeting inflammation in cancer.

In conclusion, these studies illustrate the complex cross-regulation between the Hippo/YAP and NF-κB pathways. Apparent discrepancies across studies reflect differences in tumor types, cellular models, or inflammatory contexts. YAP can promote inflammation and facilitate tumor migration and progression through NF-κB pathway activation. Conversely, YAP can reduce inflammation and exert tumor-suppressive effects by inhibiting NF-κB activity (Wang et al., 2020).

## 4 Hippo/YAP and Wnt/β-catenin pathway

The Wnt/β-catenin pathway is a well-established oncogenic signaling, which is closely associated with the progression of malignant tumors, poor prognosis, and increased cancer-related mortality (Yu et al., 2021). Functionally, it drives tumor proliferation and metastasis by promoting β-catenin nuclear translocation and its transcriptional engagement with T cell factor/lymphoid enhancer factor (TCF/LEF) factors, thereby enhancing the expression of genes associated with cell cycle progression and stemness (Song et al., 2024). Wnt pathway can be divided into three main branches, the canonical Wnt/β-catenin pathway, the non-canonical Wnt/planar cell polarity pathway (Wnt/PCP pathway), and the non-canonical Wnt/calcium signaling pathway (Wnt/Ca^2+^ pathway) (Qin et al., 2024). Interaction between the Hippo/YAP and Wnt/β-catenin pathways has been identified as a critical tumorigenic network in certain cancers (Li et al., 2019). For example, TAZ has been shown to restrict Wnt/β-catenin signaling through direct cytoplasmic interaction with Dishevelled (DVL), resulting in Wnt signaling inhibition (Varelas et al., 2010a).

Notably, emerging evidence reveals that YAP enhances β-catenin activity. In triple-negative breast cancer, receptor tyrosine kinase mesenchymal-epithelial transition factor (c-Met) induces YAP expression, and the YAP/TEAD complex subsequently interacts with β-catenin to form a YAP/TEAD/β-catenin complex. This complex binds to enhancers of Wnt target genes, promoting their transcription and modulating the oncogenic activity of triple-negative breast cancer cells (Quinn et al., 2021). In human gliomas, YAP suppresses glycogen synthase kinase 3 beta (GSK3β) activity, leading to increase the protein level and activity of β-catenin, which drives glioma cell proliferation (Wang et al., 2017). Similarly, in laryngeal and gastric cancer cells, YAP upregulates β-catenin signaling, enhancing tumor cell proliferation and invasion (Tang et al., 2019; Zeng et al., 2021). Furthermore, YAP facilitates Wnt/β-catenin-dependent proliferation of intestinal epithelial cells and plays a critical role in regulating epithelial regeneration following inflammation (Deng et al., 2022).

YAP also facilitates intestinal epithelial regeneration and inflammation resolution. In colon cancer, the hyperactivation of YAP diverts its reparative function toward tumorigenesis by triggering Wnt hyperactivity (Deng et al., 2018). This highlights a contextual switch from regenerative to oncogenic YAP functions, with Wnt crosstalk serving as a pivotal tipping point. Beyond direct activation, studies have also shown that modulating YAP expression can suppress colorectal cancer associated with aberrant β-catenin pathway activation. For instance, celastrol-induced upregulation of heat shock factor 1 (HSF1) leads to liver kinase B1 (LKB1)-mediated phosphorylation and degradation of YAP, consequently reducing β-catenin levels and suppressing colorectal cancer growth (Wang et al., 2019). These findings suggest that YAP contributes to tumor progression by activating the Wnt/β-catenin signaling pathway.

Conversely, β-catenin also reciprocally regulates YAP activity. Studies indicate that β-catenin promotes YAP nuclear translocation in melanoma-associated fibroblasts, modulating their biological functions and contributing to tumor progression (Liu et al., 2019). Similarly, β-catenin and the MYC proto-oncogene (MYC) cooperate to promote fibroblast proliferation and liver tumorigenesis through YAP activation (Bisso et al., 2020). In rectal cancer, the β-catenin/transcription factor 7-like 2 (TCF7L2) complex interacts with an enhancer element in the first intron of the YAP gene, inducing YAP expression and driving tumor progression. TCF7L2 is a high mobility group (HMG) box-containing transcription factor that plays a key role in the Wnt signaling pathway by mediating the transcription of target genes upon binding with β-catenin (Konsavage et al., 2012). Notably, a comprehensive study analyzing β-catenin-active cancer cell lines revealed that YAP expression is crucial for β-catenin-mediated carcinogenic transformation. In this study, YAP was found to form a transcriptional complex with β-catenin and T-box transcription factor 5 (TBX5), regulating the expression of anti-apoptotic genes such as Bcl-2-like protein 1 (BCL2L1) and baculoviral IAP repeat-containing protein 5 (BIRC5), thereby promoting tumorigenesis (Rosenbluh et al., 2012). YAP forms a ternary complex with β-catenin and the transcription factor TBX5. This complex associates with the promoters of the anti-apoptotic genes BCL2L1 and BIRC5, promoting their transcription and contributing to cancer malignancy and cell survival (Rosenbluh et al., 2012). These findings underscore the critical role of the β-catenin/YAP complex in tumor progression.

By contrast, evidence suggests a negative regulatory interaction between the Hippo/YAP and Wnt/β-catenin pathways. For instance, 13,14-secoergostane steroid (physalin F) enhances the interaction between YAP and the β-transducin repeat-containing protein (β-TrCP)/β-catenin destruction complex, accelerating the ubiquitination and subsequent degradation of β-catenin. As a result, physalin F inhibits the Wnt/β-catenin signaling pathway in a YAP-dependent manner, highlighting its potential as a novel therapeutic agent for colorectal cancer (Chen et al., 2018). Recent studies have further expanded on these mechanisms. For example, under conditions of inactive Wnt signaling, YAP participates in the β-catenin destruction complex by interacting with axis inhibition protein 1 (Axin1) (Azzolin et al., 2014). Within this complex, YAP recruits β-TrCP to facilitate β-catenin degradation, thereby inhibiting the Wnt signaling pathway and exerting a tumor-suppressive effect. Conversely, when Wnt signaling is active, low-density lipoprotein receptor-related protein 6 (LRP6) displaces YAP from Axin1, releasing YAP from the destruction complex (Ren et al., 2021). Additionally, YAP and TAZ, transcriptional co-activators of the Hippo pathway, suppress Wnt signaling by inhibiting β-catenin nuclear translocation, while leaving its protein stability unaffected (Zhou et al., 2011; Imajo et al., 2012). These findings highlight the complex and context-dependent roles of YAP in modulating Wnt/β-catenin signaling.

## 5 Hippo/YAP and TGF-β pathway

TGF-β is a multifunctional cytokine that plays a vital role in regulating various biological processes, including development and homeostasis. Dysregulation of TGF-β signaling is closely associated with the pathogenesis of tissue fibrosis, cancer, and other diseases (Massague, 2008). Since Ferrigno et al. first identified YAP as an interacting protein of SMAD family member 7 (Smad7) through yeast two-hybrid screening of a human placental cDNA library, increasing evidence suggests the importance of Hippo/YAP signaling in modulating disease progression (Ferrigno et al., 2002).

The Hippo pathway nuclear effectors, YAP/TAZ, interact with the TGF-β transcriptional mediators, Smads, to control Smad activity. However, it has also been reported that the Hippo/YAP signaling has both positive and negative effect in TGF-β pathway, thereby accelerating tumor progression. In primary breast tumors, YAP suppresses SMAD family member 3 (Smad3) activity, enhancing the survival and self-renewal of tumor-initiating cells, which increases the risk of breast cancer recurrence and metastasis (Sun et al., 2016). Conversely, in liver cancer cells, YAP promotes the cytoplasmic retention of Smad3, facilitating the development of tumor-initiating stem-like cells (Chen et al., 2024). In addtion, in models of breast cancer, sarcoma, and bladder cancer, YAP binds with Smad2/3 to activate different target genes such as neuronal growth regulator 1 (NEGR1), hyaluronan-mediated motility receptor (HMMR), and CTGF (Hiemer et al., 2014; Ye et al., 2020).

These differences in target gene selection may due to the subcellular localization, phosphorylation states and the downstream effect of YAP/TAZ. The Crumbs complex links cell density sensing to Hippo-mediated regulation of the TGF-β-Smad pathway, revealing a connection between Hippo signaling and TGF-β pathway control (Varelas et al., 2010b). The regulation of activator and attenuator dynamics in regulatory feedback (RAADRF) model mathematically describes dynamic feedback interactions between the YAP and TGF-β signaling pathways, demonstrating how YAP regulates Smad protein activation and attenuation in specific physiological and pathological contexts (Labibi et al., 2020). Nuclear localization of Smads is the key to TGF-β signaling. YAP does not stably bind to the core structure of the Smad complex but redirects its function by influencing Smad pairing and transcriptional persistence in specific contexts (Labibi et al., 2020). Hippo/YAP signaling can affect tumor development through TGF-β. It is important to note that TGF-β/YAP crosstalk does not always promote tumor progression. In primary breast tumors, YAP inhibits Smad3 activity, which in turn promotes the self-renewal of tumor stem cells (Sun et al., 2016). In liver cancer, YAP inhibits the nuclear translocation of Smad3, causing it to remain in the cytoplasm and inducing the generation of stem-like tumor cells (Chen et al., 2024). This functional “contradiction” suggests that the crosstalk between YAP and the TGF-β pathway is highly context-dependent. Its outcome is influenced not only by the signal components themselves but also by factors such as tumor type, tissue microenvironment, cell state, and disease progression stage. In triple-negative breast cancer, the ubiquitin-protein ligase E3 ubiquitin ligase RAD18 homolog (RAD18) activates YAP, further promoting macrophage secretion of TGF-β, forming a positive feedback loop (Yan et al., 2022). In laryngeal cancer cells, YAP enhances signal transducer and activator of transcription 3 (STAT3) activity, which induces TGF-β signaling to upregulate programmed death-ligand 1 (PD-L1) expression and promote immune evasion (Du et al., 2021).

In sarcoma cancer, TGF-β enhances YAP activity through the phosphorylation of Smad3. Phosphorylated Smad3 interacts with the YAP/TEAD1-4 complex, increasing the expression of hyaluronic acid-mediated motility receptors (HMMR/RHAMM), which facilitates the progression and metastasis of sarcoma and fibrosarcoma (Ye et al., 2020). YAP activation also facilitates the nuclear accumulation of Smad2/3, synergistically increasing the expression of CTGF, thereby contributing to the proliferation of human malignant mesothelioma (Fujii et al., 2012). Besides, suppression of the long non-coding RNA MIR497 host gene (MIR497HG) enhances YAP-Smad3 interaction, resulting in elevated CTGF transcriptional activity and accelerated bladder cancer progression (Zhuang et al., 2020). TGF-β induces Smad2/3 activation, which interacts with YAP to drive the phenotypic transition of melanoma cells from a proliferative to an invasive state (Luond et al., 2022).

In conclusion, the crosstalk between Hippo/YAP and the TGF-β pathway forms a complex and highly dynamic regulatory network. The YAP-Smad2/3 complex may cooperatively recruit different co-activators or epigenetic regulators across various cancer types, altering the transcriptional target gene profile and enabling the TGF-β pathway to switch from tumor-suppressive to tumor-promoting roles. Further exploration of this mechanism will enhance our understanding of the signaling basis behind tumor heterogeneity and provide strategic support for multi-pathway targeted interventions.

## 6 Hippo/YAP and Hedgehog pathway

The Hedgehog signaling pathway is a highly conserved mechanism that transmits signals from the cell membrane to the nucleus, playing a crucial role in regulating cell fate, proliferation, and differentiation during mammalian development. This pathway comprises Hedgehog ligands sonic hedgehog (SHH), desert hedgehog (DHH), and indian hedgehog (IHH), Patched receptors (Ptch-1 and Ptch-2), Smoothened (SMO), the kinesin family member 7 (KIF7), protein kinase A, glioma-associated oncogene family zinc finger protein (Gli) transcription factors, and the negative regulator suppressor of fused (Sufu) (Fernandez et al., 2012). Among the Gli transcription factors, Gli1 functions exclusively as a transcriptional activator, while Gli2 and Gli3 serve dual roles as both activators and repressors. Aberrant activation of the Hedgehog signaling pathway has been implicated in several cancers, including breast (Riobo-Del Galdo et al., 2019), liver (Ding et al., 2021), and prostate cancers (Suzman and Antonarakis, 2015).

As one of the core effector molecules of the Hippo pathway, YAP engages in complex signaling crosstalk with the Hedgehog pathway. During neuronal differentiation, YAP activates Hedgehog signaling pathway, enhancing the expression of downstream targets like Ptch1, thereby suppressing differentiation and promoting cell proliferation during the process (Lin et al., 2012). Overexpression of YAP can activates the Hedgehog pathway to increases the expression of the downstream target gene Ptch1 and Gli1 (Lin et al., 2012; Jeong et al., 2018). The promoted Gli1 expression by YAP enhances its transcriptional activity, thus establishing an Hh-YAP-Gli signaling loop that significantly increases the migration and proliferation of cancer cells in osteosarcoma and medulloblastoma (Gu et al., 2023). Conversely, knockdown of Gli2 facilitates neuronal differentiation even in the presence of YAP overexpression (Jeong et al., 2018). Furthermore, YAP has been identified as a downstream effector of the Hedgehog pathway, with its activation influenced by Hedgehog signaling. Inhibition of the Hedgehog pathway in hepatic stellate cells impedes YAP activation, and YAP loss suppresses Gli expression (Du et al., 2018). Additionally, knockdown of the Hedgehog co-receptor SMO reduces both messenger RNA and protein levels of YAP and activates the Hippo kinase cascade, thereby inhibiting the post-translational activation of YAP (He et al., 2021).

The interplay between the Hippo and Hedgehog signaling pathways is also implicated in tumorigenesis (Slemmons et al., 2017). Dysregulated activation of the Hedgehog pathway leads to overexpression of YAP and the long non-coding RNA H19 imprinted maternally expressed transcript, promoting the progression of osteosarcoma (Chan et al., 2014). In addition, in radio-resistant medulloblastoma, activation of the Hedgehog signaling pathway increases YAP expression. YAP confers radioresistance by driving the expression of insulin-like growth factor 2 (IGF2) and activating Akt, which allows cells to enter mitosis with unrepaired DNA (Fernandez et al., 2012).

YAP may also play a negative regulatory role downstream of the Hedgehog pathway. The expression of Hedgehog target genes depends on cell density and inversely correlates with Hippo target gene expression (Tariki et al., 2014). Overexpression of YAP inhibits Hedgehog signaling, whereas YAP knockdown enhances Gli activity within the Hedgehog pathway. Additionally, YAP has been shown to directly bind to Gli1, thereby negatively regulating its activity (Tariki et al., 2014). Despite this negative regulation, the Hedgehog signaling pathway can enhance the post-translational activity of YAP by upregulating its protein levels, creating a negative feedback loop.

The aforementioned studies highlight the complex role of the Hippo/YAP pathway in its interaction with the Hedgehog pathway. On one hand, YAP serves as a positive regulator upstream of the Hedgehog pathway, where its overexpression can activate Hedgehog signaling, inhibit neuronal differentiation, and drive oncogenesis. On the other hand, YAP exhibits bifunctional effects downstream of the Hedgehog pathway, influencing cell cycle regulation and cancer progression in both positive and negative ways. These contrasting regulatory effects suggest the presence of cell-type-specific mechanisms governing the interplay between the Hippo/YAP and Hedgehog pathways, emphasizing the need for further investigation to elucidate these precise mechanisms.

## 7 Hippo/YAP and Notch pathway

The Notch signaling pathway plays a central role in cell differentiation, development, and tumorigenesis. This pathway comprises Notch receptors, Notch ligands, and the c-promoter binding factor 1 (CBF1)/Suppressor of Hairless (Su (H))/Lin-12 and Glp-1 phenotype 1 (Lag-1) sequence. In mammals, four subtypes of Notch receptors have been identified neurogenic locus notch homolog protein (Notch-1), Notch-2, Notch-3, and Notch-4, along with five subtypes of Notch ligands, delta-like (Dll-1), Dll-3, Dll-4, Jagged-1, and Jagged-2 (Li et al., 2017). Recent studies increasingly highlight the aberrant activation of the Notch signaling pathway in various tumors. For instance, mutations in Notch pathway components have been identified in several cancer subtypes, including lung cancer (Westhoff et al., 2009), liver cancer (Xue et al., 2024), colorectal cancer (Sun et al., 2025), and breast cancer (Yousefi et al., 2022). Because Notch signaling relies on direct cell-to-cell interactions and does not use second messengers, it requires crosstalk with other signaling pathways to precisely regulate cell fate. Recent studies have revealed a wide and complex interplay between the Hippo/YAP signaling pathway and the Notch pathway. YAP not only acts as an upstream transcriptional regulator of Notch, participating in its feedback regulation, but also synergistically contributes to stem cell maintenance, tumor transformation, and immune regulation (Chen et al., 2025).

Substantial research has shown that YAP functions upstream of the Notch signaling pathway, with its overexpression leading to Notch signaling activation. The Notch ligand Jagged-1 was identified as a downstream target of YAP in hepatocellular carcinoma (Tschaharganeh et al., 2013). The study demonstrated that YAP upregulates Jagged-1 through MST1/2-and TEAD4-dependent pathways, thereby activating Notch signaling. In colon cancer cells, YAP overexpression upregulated Jagged-1, leading to activation of the Notch pathway and increased proliferation. Induction of Jagged-1, activation of Notch, and cell proliferation required binding of YAP to its transcriptional partner TEAD4, TEAD4 binding required MST1/2 but not β-catenin signaling. These findings suggest that YAP-mediated activation of the Notch pathway contributes to tumor progression in colon cancer (Tschaharganeh et al., 2013).

YAP-induced activation of Notch signaling has been observed in various cell types, including intestinal stem cells, adult liver cells, colon cancer cells, and hepatocellular carcinoma cells. In glioblastoma, the long non-coding RNA long intergenic non-protein coding RNA OIP5 (linc-OIP5) is overexpressed, and its knockout leads to decreased expression of genes in the YAP and Notch signaling pathways. Reduced YAP expression results in lower levels of Jagged-1, Notch-1, and hairy and enhancer of split-1 (Hes1) at both gene and protein levels, indicating that YAP downregulation can inhibit Notch signaling (Hu et al., 2017). In embryonic striated muscle, Notch signaling directly enhances YAP activity, leading to increased expression of the Notch ligands jagged canonical notch ligand 1 (JAG1) and DLL1 as well as the core Notch transcription factor recombination signal binding protein for immunoglobulin kappa J region (RBPJ). This reciprocal regulatory mechanism supports the development of embryonic rhabdomyosarcoma. During angiogenesis, YAP activation has been shown to suppress the expression of β-catenin and Dll-4, a Notch signaling inhibitor mediated by the Notch intracellular domain, thereby promoting vascular development (Docshin et al., 2024). In addition, bidirectional signaling mechanism between Notch and YAP has been detected (Slemmons et al., 2017). In embryonic rhabdomyosarcoma, Notch signaling directly activates YAP, which in turn enhances the expression of Notch ligands Jagged-1 and Dll-1 as well as the core Notch transcription factor RBPJ. This bidirectional interaction between YAP and Notch signaling synergistically promotes the progression of embryonic rhabdomyosarcoma (Slemmons et al., 2017).

Both YAP and Notch pathways are recognized as oncogenic drivers across various tumor types. Recent studies have shown that YAP activity can antagonize Notch function, with the biological effects of Notch signaling being highly context-dependent. Moreover, YAP has been identified as a mechanosensitive mediator of signal transduction in adult stem cells, where its activation is regulated by mechanical stimuli (Meli et al., 2023). Upon mechanical activation, YAP can bind to distal enhancers, upregulating Delta-like ligand expression. This process inhibits Notch signaling, thereby maintaining the undifferentiated state of stem cells. Conversely, YAP knockout reduces the expression of Notch ligands, facilitating the release of Notch signals and promoting epidermal cell differentiation. This finding suggests a novel regulatory mechanism in which YAP restricts tumor-initiating cells to their basal layer niches and mitigates tumorigenesis by releasing Notch signals to encourage differentiation.

## 8 Hippo/YAP and other signaling pathways

In addition to the mentioned pathways that interact with the Hippo/YAP signaling, there are still several signaling pathways, including mTOR, PD-L1, phosphatidylinositol 3-kinase (PI3K)-protein kinase B (AKT), c-jun N-terminal kinase (JNK), cyclic GMP-AMP synthase (cGAS)-stimulator of interferon genes (STING), which regulate immune microenvironment and metabolism that affect Hippo/YAP signaling during tumorigenesis. Understanding these processes provide insights into the challenges encountered in cancer treatment processes (Fernandez et al., 2012).

mTOR is a serine/threonine protein kinase, which integrates diverse extracellular and intracellular signals to regulate cell proliferation and autophagy. In various cancers, mutations in its components or hyperactivation of upstream signals can cause abnormal activation of the mTOR signaling pathway, like in breast cancer, lung cancer, and prostate cancer (Panwar et al., 2023). The constant activation of mTOR leads to uncontrolled growth and metabolic reprogramming characterized by enhanced glycolysis and amino acid uptake, which not only fuels tumor cell anabolism but also shapes the immune microenvironment (Kim et al., 2017). Therefore, mTOR inhibitors, such as rapamycin and its derivatives, are actively studied and applied in cancer treatment (Abou-Alfa et al., 2025). Interestingly, in pancreatic neuroendocrine neoplasms, YAP/TAZ overexpression activate mTOR signaling and is associated with worse prognostic outcomes (Shiraishi et al., 2025). YAP/TAZ enhance leucine uptake by inducing the expression of the high-affinity leucine transporter l-type amino acid transporter 1 (LAT1) through TEAD transcription factors. This process promotes amino acid-induced activation of mTOR complex 1 (mTORC1), giving cells a competitive growth advantage (Hansen et al., 2015). The crosstalk between Hippo/YAP and mTOR signaling pathways represents a metabolic vulnerability in cancers, highlighting opportunities for dual targeting of transcriptional and nutrient-sensing pathways to disrupt tumor progression.

Programmed cell death protein 1 (PD-1), a crucial immune checkpoint molecule, plays an important role in tumor immune escape by regulating the activity and function of immune cells. PD-L1 is an immuno-suppressive molecule that binds to the PD-1 receptor on the surface of T cells, inhibiting the activity of T cells and leading to tumor immune escape (Daassi et al., 2020; Kornepati et al., 2022). Recent studies have shown that the disruption of the Hippo/YAP signaling also shares molecular connections with immune suppression mediated by PD-L1. In uveal melanoma, YAP/TAZ directly interact with the enhancer regions of the PD-L1 gene in the nucleus, thereby activating its transcription independent of interferon-g signaling (Dong et al., 2024). In ovarian cancer, the ablation of LATS1/2, the key kinases within Hippo/YAP signaling, constitutively activates YAP/TAZ which upregulates the expression of the gene encoding PD-L1. The increased PD-L1 expression allows tumor cells to more effectively evade the immune surveillance of T cells (Zhu et al., 2025).

Moreover, the cGAS-STING signaling pathway interacts with Hippo/YAP signaling, significantly influencing tumor immunity and progression (Hao, 2022). The cGAS-STING pathway serves as a primary sensor for cytosolic DNA, initiating type I interferon responses and enhancing anti-tumor immunity (Suter et al., 2021). Studies have demonstrated that activation of cGAS-STING signaling pathway can suppress YAP/TAZ activity, thereby inhibiting tumorigenesis (Wang and Xing, 2025). For instance, in non-small cell lung cancer, loss of cGAS-STING signaling leads to increased YAP/TAZ activity, promoting tumor immune evasion and progression (Hao, 2022). Additionally, YAP can regulate the expression of cGAS through interactions with the Notch1 signaling pathway, further modulating STING-mediated inflammatory responses (Zhang et al., 2017). These findings underscore the complex interplay between cGAS-STING and Hippo/YAP signaling pathways, suggesting that combined targeting of these pathways may offer promising therapeutic strategies in cancer treatment.

Furthermore, the YAP/TEAD complex directly promotes the transcription of insulin receptor substrate 2 (IRS2), which subsequently activates phosphatidylinositol 3-kinase (PI3K)/AKT pathway in medulloblastoma (Fernandez et al., 2012). Specifically, IRS2 recruits and activates PI3K, which phosphorylates phosphatidylinositol (4,5)-bisphosphate (PIP2) to generate phosphatidylinositol (3,4,5)-trisphosphate (PIP3). PIP3 then facilitates the membrane localization of AKT and its upstream kinase PDK1, enabling PDK1 to phosphorylate AKT at Thr308. Full activation of AKT is further achieved via phosphorylation at Ser473 by mTORC2 (Taniguchi et al., 2006; Hushmandi et al., 2025). This signaling cascade accelerates tumorigenic potential and enhances resistance to radiation (Fernandez et al., 2012). The JNK signaling pathway, also termed stress-activated protein kinase (SAPK), is a pivotal component of the mitogen-activated protein kinase (MAPK) family. It critically regulates cellular processes including proliferation, apoptosis, differentiation, tumorigenesis, and immune responses (Wagner and Nebreda, 2009). Emerging evidence highlights significant crosstalk between the Hippo/YAP and JNK pathways (Liu et al., 2016). For example, a study shows that dysfunction of the Hippo/YAP pathway activates JNK via transcriptional upregulation of Rho1, a process linked to tumorigenesis (Ma et al., 2015). Within the JNK cascade, AP-1 functions as a pivotal transcription factor complex composed of JUN and FOS family proteins, whose transcriptional activity is enhanced by JNK-mediated phosphorylation (Jaiswal et al., 2023). This activation regulates diverse cellular processes, including proliferation, differentiation, apoptosis, inflammation, migration, and invasion. Notably, YAP-driven cell proliferation has been shown to be highly dependent on functional AP-1 (Koo et al., 2020). YAP can activate the JNK/AP-1 axis to promote hepatocyte proliferation, thereby contributing to hepatic enlargement and tumorigenesis (Koo et al., 2020). In triple-negative breast cancer, AP-1 family member JUNB and STAT3 cooperatively recruit YAP, which acts as a transcriptional co-activator for both complexes, thereby enhancing oncogenic transcriptional outputs and correlating with poor clinical outcomes (He et al., 2021).

The complex network of intracellular signaling pathways is marked by extensive crosstalk, with recent studies highlighting interactions between the Hippo pathway and various signals involved in tumorigenesis. A deeper understanding of the mechanisms and effects of Hippo pathway interactions with other signaling pathways, such as JNK and AP-1, could offer valuable insights for developing new therapeutic approaches for inflammation, cancer, and related diseases.

Collectively, while numerous signaling pathways, including mTOR, PD-L1, PI3K/AKT, JNK, cGAS-STING, and AP-1 interact with Hippo/YAP signaling and influence tumorigenesis, this review focuses on six major pathways: NF-κB, Wntβ/Catenin, TGF-β, Hedgehog, Notch, and the core Hippo/YAP pathway. These pathways were selected due to their well-characterized and recurrent interactions with Hippo/YAP across multiple cancer types, the abundance of high-quality studies available in recent years, and the existence of FDA-approved drugs or clinical trials exploring combination therapies involving these axes. Such emphasis allows for a deeper mechanistic exploration and a more translationally relevant discussion, especially regarding potential therapeutic strategies.

## 9 Prospect

**FIGURE 2:**
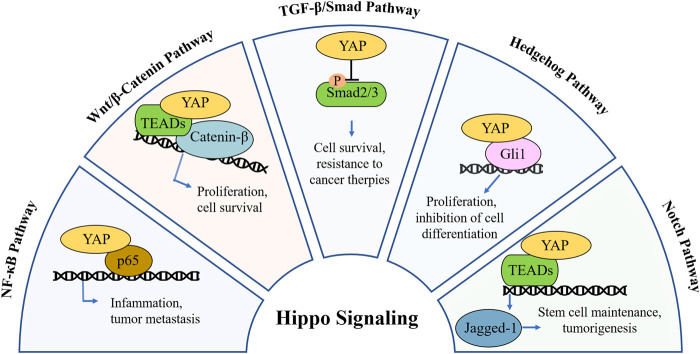
Mechanistic crosstalk between YAP and other pathways in cancer. In NF-κB pathway, YAP acts as a p65 co-activator or competitor, modulating inflammation and metastasis. In Wnt/β-catenin pathway, YAP forms complex with β-catenin to drive or suppress Wnt target genes, modulating tumor cell proliferation and survival. In TGF-β pathway, YAP binds with Smad2/3, altering transcription or nuclear retention, modulating cell survival and linked with resistance to cancer therapies. In Hedgehog pathway, YAP regulates Gli1 activity and its nuclear localization, inhibiting cell differentiation and modulating tumor cell proliferation. In Notch pathway, YAP modulates Jagged-1, promoting or limiting Notch signaling in a context-dependent manner, regulating cancer stem cell maintenance and tumorigenesis.

The Hippo/YAP signaling plays a crucial role in regulating cell proliferation, maintaining tissue homeostasis, and contributing to tumorigenesis. Recent research on pathways such as NF-κB, TGF-β, and Wnt/β-catenin suggests that the Hippo pathway does not function in isolation in cancer. Instead, it interacts with these pathways, mediated primarily through the effector protein YAP, forms a complex regulatory network governing cellular growth and oncogenesis (Figure 2). For example, YAP can activate the NF-κB pathway by suppressing ubiquitin specific peptidase 31 (USP31) expression (Philot Pavao et al., 2018), while simultaneously dampening NF-kB activity via interactions with the TAK1/IKK complex (Bulaklak et al., 2018). This dual regulation highlights the complexity of signaling networks and explains the limitations of single-target therapy in cancer treatment.

Given these challenges, oncology research has increasingly focused on developing Hippo-targeted therapies, including novel small-molecule inhibitors, advanced drug delivery systems and combination strategies. Several innovative drugs targeting the Hippo/YAP signaling have successively entered the early-stage clinical trial phase in recent times (Chapeau et al., 2024). These drugs have exhibited remarkable anti-tumor activity during the trials, which has triggered extensive attention and anticipation. They are expected to bring about new breakthroughs and transformations in the field of cancer treatment (Table 1). Innovative drug delivery systems, such as nanoparticles and ligand-targeted carriers, are engineered to enhance the bioavailability and tumor-specific accumulation of Hippo/YAP-targeted agents. Specifically, encapsulating these drug within liposomes or biomimetic nanoparticle has been shown to improve therapeutic efficacy by overcoming biological barriers and enabling precise drug delivery (Qi et al., 2024).

**TABLE·1:** Recent Hippo-targeting agents in clinical trials.

Agent	Type	Mode of action	Clinical trial	Reference/Trial Number
Verteporfin	Small-molecule inhibitor	Blocks YAP-TEAD interaction	Phase 1-2	NCT04590664
IK-930	Small-molecule inhibitor	TEAD inhibitor	Phase 1	NCT05228015
BPI-460372	Small-molecule inhibitor	TEAD inhibitor	Phase 1	NCT05789602
VT3989	Small-molecule inhibitor	TEAD inhibitor	Phase 1	NCT04665206
IAG933	Small-molecule inhibitor	Blocks YAP-TEAD interaction	Phase 1	NCT04857372 PMID: 38886525 (Chapeau et al., 2024)
Norcantharidin	Demethylated derivative	Affects tumor cell processes in Hippo pathway	Pre-clinical	PMID: 27903989 (Guo et al., 2017)
Rottlerin	Natural product	Inhibits Hippo-related kinases	Pre-clinical	PMID: 27999199 (Zhao et al., 2017)

In addition, combination therapies pairing Hippo pathway inhibitors with mTOR blockers, PD-L1 inhibitors, or chemotherapeutics address single-agent resistance by targeting interconnected oncogenic and immune escape mechanisms. Preclinical studies show these combinations synergistically reverse immunosuppressive tumor microenvironments and eliminate therapy-resistant cells. For example, the combination of Verteporfin and anti-PD-L1 enhances T cell infiltration in ovarian cancer through blocks YAP-mediated PD-L1 upregulation (Zhu et al., 2025). While IK-930 combined with everolimus reduces proliferation in PI3K-driven sarcomas at low doses, with *in vivo* tumor growth inhibition (Garcia et al., 2025). A recent analysis (Evsen et al., 2023) identifies broad synergy between TEAD inhibitors and multiple drug classes like immune checkpoint and mTOR inhibitors, likely by suppressing compensatory pathways activated during Hippo/YAP suppression. These efforts not only deepen our understanding of Hippo pathway biology but also accelerate the translation of precision oncology for improved patient outcomes.

This review explores the composition of the Hippo pathway and the regulatory role of its core component, YAP, within the broader signaling network. The aim is to enhance understanding of the Hippo pathway as a central hub in cellular signaling and to provide valuable insights for the development of personalized cancer therapies. Further investigation into the interactions between the Hippo pathway and other signaling pathways represents a promising and significant avenue for advancing cancer research and treatment strategies.
